# Proposed observational study protocol for early differentiation of cytokine release syndrome and sepsis in CAR-T recipients with haematological malignancies using the IL-6/PCT ratio: the DRACARYS study

**DOI:** 10.3389/fonc.2025.1683350

**Published:** 2025-11-19

**Authors:** Anas Ibraheem, Melanie Dalby

**Affiliations:** 1 Haematology Department, King’s College Hospital NHS Foundation Trust, London, United Kingdom; 2 King’s College London School of Cancer & Pharmaceutical Sciences, London, United Kingdom

**Keywords:** CAR-T therapy, haematological malignancies, cytokine release syndrome, sepsis, biomarkers, IL-6, PCT, tocilizumab

## Abstract

Chimeric Antigen Receptor T-cell (CAR-T) therapy has revolutionised treatment for haematological malignancies, demonstrating remarkable efficacy in B-cell leukaemias, lymphomas, and multiple myeloma. However, severe toxicities—particularly Cytokine Release Syndrome (CRS) and sepsis—present significant clinical challenges. Both conditions share overlapping features, including fever, hypotension, and multi-organ dysfunction, making timely and accurate differentiation essential. CRS is driven by excessive cytokine release, predominantly IL-6, and is treated with IL-6 receptor blockade (tocilizumab) and corticosteroids. Sepsis, by contrast, results from a dysregulated immune response to infection and requires antibiotics, as well as supportive care. Due to diagnostic uncertainty, clinicians often treat both conditions empirically. This can lead to inappropriate therapies—immunosuppressives may worsen sepsis, while antibiotics in CRS contribute to antimicrobial resistance and unnecessary healthcare burden. Existing biomarkers, such as IFN-γ and IL-1β, have shown potential but are limited by cost, availability, and the lack of rapid bedside implementation. There is a pressing need for a clinically accessible and reliable biomarker to distinguish CRS from sepsis in CAR-T patients. We hypothesise that the IL-6/procalcitonin (PCT) ratio will improve diagnostic accuracy. IL-6 is elevated in both conditions, while PCT is more specific to bacterial infection. However, PCT alone may be unreliable in immunocompromised patients, such as those receiving CAR-T therapy. The IL-6/PCT ratio is expected to reduce inter-individual variability and address limitations inherent to each marker when used alone. In this multi-centre, observational, prospective study, we will evaluate the IL-6/PCT ratio in febrile CAR-T patients. The primary analysis will focus on relapsed/refractory B-cell lymphomas, with a prespecified expansion/validation across other CAR-T indications. Clinical adjudication will serve as the standard of reference. We will assess diagnostic performance using Receiver Operating Characteristic (ROC) analysis to determine sensitivity, specificity, and optimal cutoffs. This study, titled *DRACARYS* (Differentiating Reactions—CRS versus sepsis—After CAR-Ts), aims to enhance diagnostic precision, guide timely and appropriate treatment, and reduce complications and unnecessary healthcare utilisation in CAR-T recipients.

## 1 Introduction

Chimeric Antigen Receptor T-cell (CAR-T) therapy represents a groundbreaking advancement in treating haematological malignancies, demonstrating exceptional efficacy and long-term clinical benefits (1). CARs are engineered synthetic receptors designed to redirect lymphocytes, primarily T cells, to identify and eliminate target antigen-expressing cells, independent of Major Histocompatibility Complex (MHC) presentation. This mechanism facilitates robust T-cell activation and potent anti-tumour responses (2). Currently, CAR-T therapies target the B-cell maturation antigen (BCMA) for multiple myeloma (MM) and the B-cell antigen CD19 for B-cell leukaemias and lymphomas. The success of CAR-T therapy in haematologic cancers has stimulated growing interest in expanding its application to other malignancies (3–6).

However, despite its success, CAR-T therapy is associated with severe toxicities that may compromise efficacy and pose life-threatening risks, such as multiple organ dysfunction, sepsis, Immune Effector Cell-Associated Neurotoxicity Syndrome (ICANS), and disseminated intravascular coagulation (DIC) (7, 8). Among these, Cytokine Release Syndrome (CRS) is the most prevalent and severe complication, characterized by an excessive systemic inflammatory response driven by hyperactivated CAR-T cells and other immune cells, such as macrophages and dendritic cells. CRS symptoms, including fever, hypotension, hypoxia, and multiorgan failure, often overlap with sepsis, making differentiation between the two conditions clinically challenging (1, 9, 10). Given the limitations of current severity grading systems that rely primarily on clinical manifestations, there is a critical need for improved diagnostic tools that enable early and precise differentiation between CRS and sepsis.

Pathophysiologically, CRS is predominantly mediated by IL-6, making IL-6 receptor blockade with tocilizumab the frontline treatment, often complemented by corticosteroids (11–13). However, severe CRS cases, particularly those complicated by secondary hemophagocytic lymphohistiocytosis/macrophage activation syndrome (HLH/MAS), may be refractory to IL-6 inhibition and require more aggressive immunosuppressive interventions, including chemotherapy (1, 14, 15). While CRS has been extensively studied, distinguishing it from sepsis remains an unresolved challenge. Unlike CRS, sepsis arises from a dysregulated immune response to infection and is primarily managed with broad-spectrum antibiotics and supportive care rather than cytokine blockade. Importantly, misdiagnosing CRS as sepsis or vice versa can lead to inappropriate treatment strategies, as immunosuppressive therapies such as IL-6 inhibitors and corticosteroids may exacerbate underlying infections. At the same time, unnecessary antibiotic use in CRS patients may contribute to antibiotic resistance and adverse effects (9, 10, 16, 17). Studies have found that 23–42% of patients developed infections within the first month after CAR-T therapy, with 31% affected between days 31 and 180 (18, 19).

Recognising the urgent need for precise diagnostic criteria, multiple biomarker-based models have been proposed to differentiate CRS from sepsis. The first model, incorporating IFN-β, CXCL1, and CXCL10, demonstrated remarkable performance, with sensitivity and specificity of at least 90% in both the training and validation cohorts. Additionally, a more complex five-cytokine model (CXCL10, CCL19, IL-4, VEGF, and CCL20) demonstrated similarly high sensitivity (91.67% training, 95.65% validation) and specificity (98.44% training, 100% validation). These robust and reliable models hold significant potential to enhance the early detection of infections during immune therapy, thereby enabling timely and appropriate intervention (20).

Studies have identified cytokine profiles, such as IFN-γ and IL-1β, as potential biomarkers for distinguishing CRS from sepsis. For instance, Diorio et al. developed a high-accuracy classification model utilizing IFN-γ and IL-1β in critically ill paediatric patients (11). Luo et al. previously introduced a diagnostic approach combining the “double peaks of IL-6” pattern with a three-cytokine-based prediction model to rapidly identify severe infections following CAR-T cell infusion (21).

However, the above-proposed models present certain limitations, including challenges in clinical implementation, a lack of rapid and widely accessible diagnostic assays, and high costs that reduce feasibility in real-world settings. Although current CAR-T therapy strategies—including low-intensity protocols, reduced-intensity lymphodepletion, and modified infusion approaches—have been developed to minimise toxicity in high-risk patients and reduce the incidence of CRS and sepsis, these complications remain significant. Notably, the risk has not been eliminated, and distinguishing between CRS and sepsis remains clinically crucial (9, 22, 23). Given these limitations, there is a pressing need for a practical, bedside evaluation tool to support early diagnosis and guide timely, appropriate management in routine clinical practice.

While CAR-T therapy was first approved for haematological cancers, its applications have rapidly expanded to solid tumours and autoimmune diseases.Recent reviews document hundreds of ongoing clinical trials in solid tumours, addressing challenges such as antigen heterogeneity and suppression of the tumour microenvironment. Simultaneously, CAR-T is being actively piloted in autoimmune conditions, including systemic lupus erythematosus, myasthenia gravis, systemic sclerosis, neuromyelitis optica, and multiple sclerosis, with early studies showing encouraging safety and efficacy profiles (24). As CAR−T expands across oncology, immunology, rheumatology, and beyond, the capacity to distinguish CRS from sepsis becomes even more critical. A reliable bedside differentiation tool would therefore benefit a broad spectrum of patients across disciplines, not just those in haemato-oncology.

A comparative review of cytokine profiles in paediatric ICU patients showed that, although the median IL−6 level was higher in CRS than in sepsis, IL−6 elevations overlapped significantly between the two groups; thus, using IL−6 alone can’t reliably distinguish the two (10). Although PCT is more specific to bacterial infections (25), studies have shown that PCT may produce false negatives, particularly in early localised infection, immunosuppression, or neutropenia, where systemic elevations are blunted. Accordingly, the diagnostic utility of PCT in neutropenic patients remains uncertain. In a retrospective series of 273 febrile neutropenic episodes in haematologic malignancy, PCT showed 46.9% sensitivity and 83.3% specificity at the 0.5 ng/mL cut-off (26). Another retrospective study of 98 CAR-T recipients for haematological malignancies found that while elevated PCT (>0.4 ng/mL) correlated with infection, its diagnostic performance was modest (AUC 0.62), underscoring limited sensitivity in this context (27). However, these findings support the rationale that PCT alone may be unreliable in CAR-T patients, reinforcing the potential for a combined biomarker strategy with IL-6.

We hypothesise that the IL-6/PCT ratio, as a novel biomarker, will improve diagnostic accuracy in distinguishing sepsis from CRS in CAR-T recipients. PCT provides an infection-specific signal, while IL-6 reflects systemic inflammatory burden; their ratio is expected to reduce inter-individual variability and address limitations inherent to each marker when used alone (28, 29). Given the absence of established cutoffs for the IL-6/PCT ratio, we will adopt an exploratory approach to this analysis. This model will be applied prospectively in febrile CAR-T patients across multi-centre cohorts to ensure clinical relevance. Although no prior studies have defined diagnostic thresholds, we will assess the ratio’s performance using Receiver Operating Characteristic (ROC) curve analysis, with clinical adjudication of sepsis versus CRS as the reference standard. This will enable us to determine sensitivity, specificity, and optimal cutoffs, providing the first data-driven foundation for its clinical application. Ultimately, this study, titled DRACARYS by the authors (Differentiating Reactions—CRS versus sepsis—After CAR-Ts), aims to enhance diagnostic precision, guide appropriate treatment, and reduce both clinical complications and unnecessary healthcare expenditures.

## 2 Study objectives

This study evaluates the IL-6/PCT ratio as a biomarker to distinguish CRS from sepsis in patients with haematological malignancies receiving CAR-T therapy. The primary focus is relapsed/refractory (R/R) B-cell lymphomas, with prespecified validation in other CAR-T indications. The aim is to enable real-time clinical decision-making and improve patient management by narrowing the diagnostic gap between CRS and sepsis. Our specific objectives are:

### 2.1 Primary objective

To evaluate the diagnostic performance of the IL-6/PCT ratio for distinguishing CRS from sepsis in adult CAR-T recipients, derive data-driven cut-offs in the primary lymphoma cohort.

### 2.2 Secondary objectives (exploratory)

1. To describe the temporal dynamics of IL-6, PCT, and the IL-6/PCT ratio during days 0–14 post-CAR-T infusion.
2. To externally validate the fixed lymphoma cut-off for the IL-6/PCT ratio in a prespecified expansion cohort of B-cell acute lymphoblastic leukaemia (B-ALL) and multiple myeloma (MM).
3. To explore the relationship between biomarker profiles and key short-term outcomes, including ICU admission, requirement for vasopressors, and 30-day mortality.

## 3 Study methodology

### 3.1 Study design

**Figure 1:**
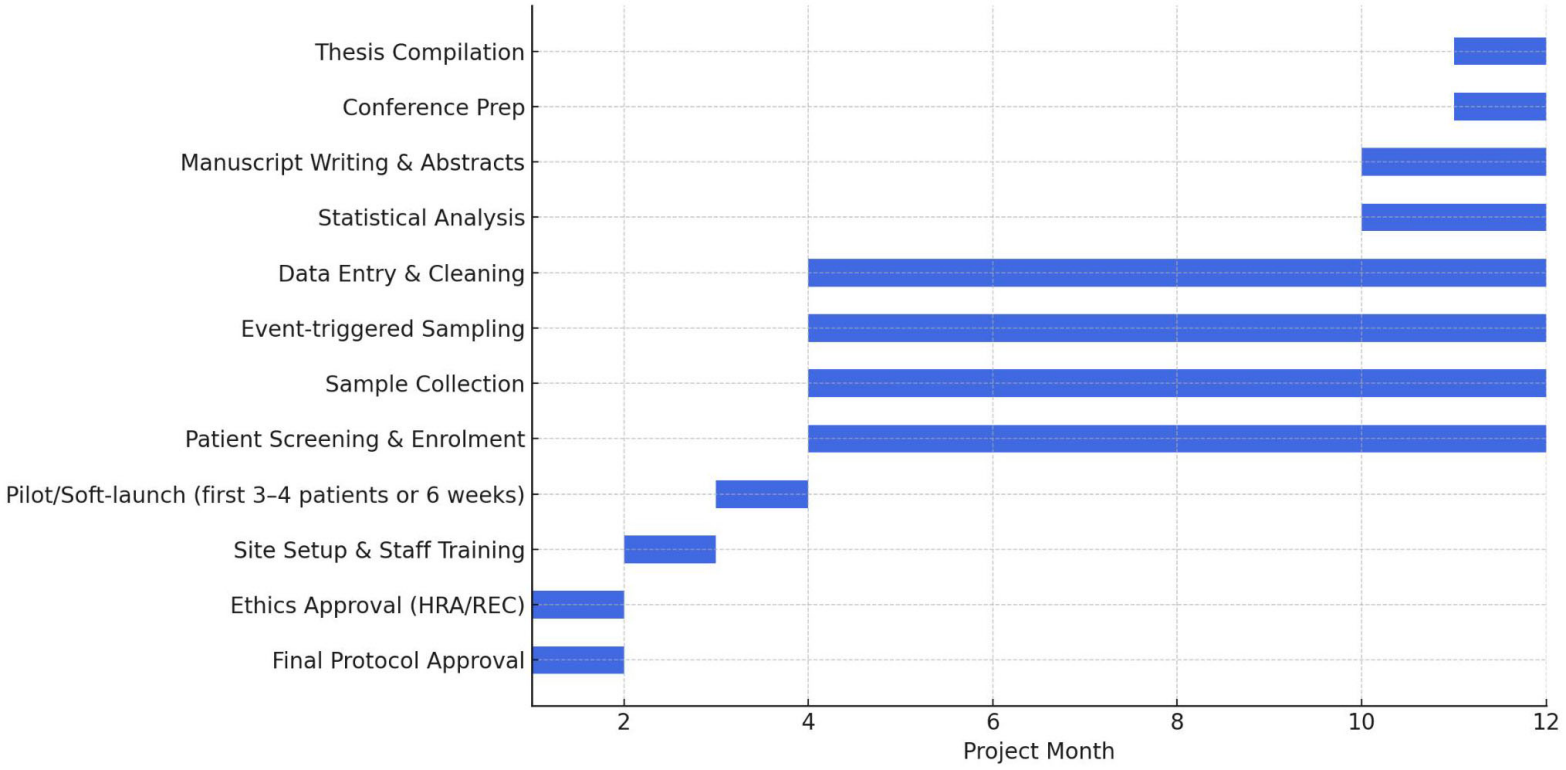
Gantt chart timeline for the DRACARYS study (12-month project plan).

- Type: Prospective exploratory observational cohort Study. IL-6/PCT measurements will not influence management during observation; all care will follow standard practice.
- Study sites: Multicentre study conducted across tertiary CAR-T treatment centres. The manuscript serves mainly as a proposal outlining a conceptual framework that could be applied nationally and/or internationally.
- Duration: 12–18 months. The overall project timeline, including patient recruitment, sample collection, data analysis, and dissemination activities, is illustrated in **Figure 1**.

- Pilot study: A short rolling pilot phase will be conducted prior to full recruitment to stress-test operational aspects of the study, including the timing and feasibility of sampling, laboratory workflows, feasibility of the clinical adjudication process, and the functionality of the electronic case report forms (eCRFs) within the Research Electronic Data Capture (REDCap) database and associated data entry processes. It is worth noting that two independent clinicians will classify febrile episodes as CRS/sepsis, and a third adjudicator will resolve any discrepancies.

The pilot phase will include the first 3–4 consecutive eligible patients or a maximum of 6 weeks, whichever occurs first. These participants will be fully consented and recruited under the same approved protocol, and will be included in the final analysis dataset unless a substantive protocol amendment is made.

Continuation to full recruitment will proceed if the following criteria are met:

1. At least 80% of planned samples are successfully obtained.
2. Laboratory assay turnaround times are within acceptable site standards.
3. No significant protocol deviations are observed.
4. Blinding target: ≥90% of fever episodes adjudicated under maintained blinding to IL-6/PCT.

- The proposed ratio (IL-6/PCT) will be monitored over 14 days (from pre-CAR-T to D14 post-infusion) at multiple sampling times, which will be discussed in detail in the later paragraphs. We will follow IL-6 and PCT for 14 days to capture infections that often arise after the first week post–CAR-T, when CRS predominates; this extended window reduces time-related confounding and misclassification, yielding less biased accuracy estimates (17, 30–32). It is worth noting that no treatment or intervention decision will be made based on this ratio during its observation. However, the patient’s treatment will be guided purely by the current evidence-based practice and local guidelines. Therefore, in this observational exploratory study, it is not necessary to obtain the biomarker results at specific time points. However, we will analyse the data retrospectively, using results as they become available in the database, with clinical adjudication of sepsis versus CRS as the reference standard. Before lymphodepletion for CAR-T, all eligible patients, as defined by the inclusion/exclusion criteria detailed in **Table 1**, will provide consent. We will enrol a primary analysis cohort of adults with relapsed/refractory B-cell lymphomas receiving CD19-directed CAR-T. To preserve feasibility and external relevance, we will also enrol a pre-specified expansion/validation cohort of other CAR-T recipients (e.g., B-ALL, BCMA-targeted myeloma) under identical sampling and laboratory procedures.

**Table 1:** Inclusion and exclusion criteria.

Inclusion criteria	Exclusion criteria
Primary analysis cohort (disease-specific) Adults (≥18 y) with relapsed/refractory B-cell lymphomas receiving CD19-directed CAR-T (e.g., Large B-cell Lymphomas subtypes, including Diffuse/high-grade B-cell lymphoma/transformed follicular lymphoma; ± mantle-cell lymphoma if using CD19 CAR-T). Baseline Eastern Cooperative Oncology Group (ECOG) performance status (0–4). Develop fever ≥38°C within 14 days post-infusion. Provide informed consent.	Pre-existing active infection, fever, or on antibiotics before CAR-T infusion (except prophylactic antimicrobials per institutional policy). Recent use of tocilizumab or high-dose steroids before CAR-T infusion. Active autoimmune or inflammatory conditions.
		Expansion/validation cohort (for feasibility/external relevance) Adults with other CAR-T indications (e.g., B-ALL receiving CD19 CAR-T or multiple myeloma receiving BCMA CAR-T) under identical sampling and lab procedures; used for external validation and, if numbers permit, secondary pooled analyses.

### 3.2 Data collection and biomarker monitoring

- Baseline demographics and clinical covariates (age, sex, ethnicity, type of malignancy, lymphodepletion regimen before CAR-T, CAR-T product, underlying chronic diseases).
- Fever pattern (duration, peak temperature, antipyretic response).
- Microbiology (Blood/urine/respiratory/other cultures, PCRs, and antigens) draw time, positivity, organism, and time to positivity.
- Organ dysfunction parameters (Sequential [Sepsis-related] Organ Failure Assessment (SOFA) score, oxygen requirement, haemodynamic instability). SOFA components will be recorded at least daily or whenever there is a clinical change. When primary measures are unavailable, we will use validated surrogates: SpO_2_/FiO_2_ may substitute for PaO_2_/FiO_2_ in the respiratory component; AVPU (Alert, Voice, Pain, Unresponsive) to GCS (Glasgow Coma Scale) mapping is permitted for the CNS component (A = 15, V = 12, P = 8, U = 3). The cardiovascular component will be determined by vasopressor presence/dose; renal (creatinine or urine output), liver (bilirubin), and coagulation (platelets) will follow Sepsis-3 definitions (33–35). If any component is unavailable within the window, it will be recorded as missing; we will report a partial SOFA (sum of available components) with an “incomplete” flag. *(SOFA is not part of the primary endpoint and, if used analytically, serves only as a pre-specified covariate in secondary models.*.
- Daily routine laboratory data (e.g., complete blood count, CRP, ferritin, fibrinogen, LDH, lactate).
- Interventions received (Tocilizumab, corticosteroids, antibiotics, vasopressors).
- IL-6: key driver of CRS; highly elevated in CRS but also present in sepsis (10).
- PCT: specific marker of bacterial infection; significantly increased in sepsis but low in CRS unless secondary infection occurs and can give false values for infections in cases of early localised infection, immunosuppression, or neutropenia, where systemic elevations are blunted (25–27).
- IL-6/PCT: the ratio reduces inter-individual level differences.
- All scheduled D0–D14 samples will be taken alongside standard-of-care bloods where feasible. Additional pre-tocilizumab/steroid and event-triggered samples may require a separate draw; drawn volume will be the minimum necessary for IL-6 and PCT assays.

We would consider the potential effects of treatment for presumptive CRS (tocilizumab and/or corticosteroids), which can affect the interpretation after receiving such treatment. Tocilizumab may transiently elevate circulating IL-6 by blocking receptor-mediated clearance, whereas corticosteroids suppress both, but exclusively IL-6 levels through broad immunosuppression (36, 37). A transparent, instructive blood sampling strategy will be adopted to mitigate these confounding factors. Core plan: blood samples will be collected at baseline (Day 0, pre-CAR-T) and once daily from Day 1 to Day 14. In patients receiving tocilizumab or corticosteroids, an additional sample will be collected immediately before drug administration to avoid pharmacologic distortion of cytokine and/or PCT levels, as mentioned in **Table 2**. In cases of acute clinical deterioration outside scheduled draws, an event-triggered sample may be obtained at the physician’s discretion. The latter can be crucial in instances where fever is not present, despite other signs of CRS/sepsis (38, 39), including haemodynamic instability (hypotension, tachycardia, altered mental status, low urine output (<0.5 mL/kg/hr), cool and clammy skin, and elevated lactate levels).

**Table 2:** Sampling and clinical timeline for biomarker measurement.

Day	Event	Biomarker collection	Notes
D0	CAR-T infusion (baseline)	Yes (once)	Pre-infusion baseline biomarkers.
D1–D14	Post-infusion monitoring	Once daily^1^	Routine collection; timing recorded.
Tocilizumab/steroids (any day)	Before administration	Yes (extra sample)^2^	Sample drawn just before immunosuppressive therapy; Draw immediately before the dose.
Event-triggered (any day)	Clinical deterioration	Yes (at discretion)^3^	If new/worsening features suggest CRS or sepsis.

^1^If a sample is collected within 2 hours of the scheduled routine draw (either ≤2 h before or ≤2 h after), do not perform an additional routine sample. Count that sample as the daily draw and proceed with the next scheduled time-point. Applies equally to event-triggered and pre-immunomodulator samples; pre-dose timing takes precedence when both apply.

^2^Ideally, take the sample before rescue immunomodulation; do not delay urgent care.

^3^Do not add an extra interim sample during a continuous febrile period. Take an additional sample only if the patient has been afebrile ≥12 h, or immediately before a planned immunomodulator dose. If another tocilizumab/steroid dose is scheduled within 6–8 h, defer to the next pre-dose sample.

### 3.3 Laboratory protocols for IL-6 and PCT assays

IL-6 and PCT concentrations will be measured using standardized, quality-assured immunoassays according to the manufacturers’ recommendations. IL-6 will be quantified on Roche Elecsys^®^ IL-6 (electrochemiluminescent immunoassay); Turnaround Time (TAT) ~18 minutes; limit of detection (LoD) ~1.5 pg/mL; functional measuring range to ~5000 pg/mL (per IFU) (40, 41). PCT will be measured on B·R·A·H·M·S^®^ PCT (KRYPTOR) using the TRACE immunofluorescent method; TAT ~19 minutes; LoD 0.02 ng/mL; wide reportable range with on-board auto-dilution (the site-verified upper reportable limit will be documented in the laboratory SOP) (42). Serum will be separated within 60 minutes of venepuncture (centrifugation 1500 × g, 10 minutes) and stored at −80°C; ≤1 freeze–thaw cycle is permitted. All assays will be conducted in accredited institutional laboratories, following the International Organization for Standardization (ISO) 15189 guidelines, to ensure analytical validity and reproducibility across participating sites. To ensure comparability, PCT values reported in ng/mL (μg/L) will be converted to pg/mL (×1000) prior to ratio calculation; the IL-6/PCT ratio will therefore be dimensionless and defined as IL-6(pg/mL) ÷ PCT(pg/mL). All diagnostic cut-offs and ROC analyses will utilise this harmonised definition, with log-transformed values employed in the regression analysis.

### 3.4 Clinical adjudication framework for CRS vs sepsis

- Adjudication panel and blinding: Each febrile episode will be independently adjudicated by two clinicians (one haematologist and one infectious diseases/critical care physician), who will be blinded to IL-6 and PCT values obtained for research purposes. A third adjudicator will resolve disagreements. Adjudicators will have access to all other clinical data, including vital signs, laboratory tests (excluding IL-6/PCT), cultures, imaging, administered therapies, and the clinical course.
- Episode definition: the first qualifying febrile event (temperature ≥38.0°C) occurring within days 0–14 after CAR-T infusion (or a clinically significant deterioration suggestive of CRS/sepsis without fever). The episode window spans 48 hours before the index timepoint (t_0_) to 14 days after (t_0_+14 days) and captures signs/symptoms, microbiology, imaging, organ dysfunction (SOFA), rest of the blood results, and treatments. A new episode is recorded only if there is ≥48 hours afebrile (or clearly resolved syndrome) plus a new diagnostic work-up/source; otherwise, events are considered part of the same episode. For the primary analysis, one index episode per patient is included. Additional episodes (if any) may be analysed in secondary or sensitivity analyses.

#### 3.4.1 Operational definitions (pre-specified)

- CRS will be defined and graded according to the American Society for Transplantation and Cellular Therapy (ASTCT) consensus criteria (43). Fever ≥38.0 °C temporally related to CAR-T infusion with compatible features (hypotension, hypoxia), without a confirmed alternative infectious source.
- Sepsis will be defined according to the Third International Consensus Definitions for Sepsis and Septic Shock (Sepsis-3) (33): A suspected or documented infection with acute organ dysfunction represented by an increase in SOFA score ≥2 points from baseline.
- Evidence of infection includes one or more of:
- Positive culture from a normally sterile site with a compatible syndrome.
- Concordant non-sterile site culture(s) plus imaging/clinical evidence of a source.
- Clear radiologic source (e.g., new lobar consolidation on chest x-ray or CT-scan) with compatible clinical course.
- Clinically compelling infection; only when cultures are negative/pending and imaging is absent/non-diagnostic. For instance, site-specific symptoms/signs with objective corroboration, such as undertaking source control (e.g., drainage, removal of peripheral or central lines, debridement). Because antibiotics are started empirically at the first fever spike, their use alone is not evidence of infection in febrile CAR-T patients (see Introduction).

### 3.5 Standardised adjudication checklist

Every episode is evaluated using the same information, ensuring consistent and auditable decisions.

- Timing (day post–CAR–T; time since lymphodepletion).
- Vitals/organ support (max temperature, vasopressors, O_2_/ventilation, lactate; SOFA score).
- Microbiology (cultures with timestamps, PCRs, and antigens).
- Imaging (CXR/CT/US, dates).
- Source control (if any): Drainage/line removal/debridement.
- Therapies (antibiotics, tocilizumab/steroids with dose/times).
- High infection-risk confounders (ANC < 0.5 × 10^9/L, mucositis, central line, recent procedures).
- Adjudication category + rationale: Final label (CRS, Sepsis, overlap, etc.) with a one-line reason—gives transparency.

### 3.6 Quality assurance

- Inter-rater agreement (Cohen’s κ, 95% CI): Two independent clinicians (haematology and ICU/infectious diseases) adjudicate each episode, blinded to IL-6/PCT levels. We will compute Cohen’s κ with 95% CIs on the initial independent ratings (before tie-break) and also report percent agreement and brief reasons for discordance. Interpretation: κ≈0.61–0.80 substantial, κ>0.80 almost perfect. An interim quality assurance/statistics summary will be produced after the first 10 episodes, and a final report will be generated at study close.
- Calibration exercise (first 5–10 episodes): The adjudicators will review the first cases together to align rule application and update a short adjudication guide. If κ<0.60 at interim review, a brief recalibration will be performed and κ re-assessed on the next 10 episodes.

#### 3.6.1 Adjudication categories (with evidence levels)

- Definite CRS: Meets ASTCT criteria; no microbiologic/radiologic evidence of infection within the episode window; alternative causes excluded.
- Probable CRS: Meets ASTCT criteria; infection evaluation negative or non-diagnostic; no organ dysfunction attributable to infection.
- Definite Sepsis: Meets Sepsis-3 with microbiologic confirmation (sterile-site culture) and compatible source.
- Probable Sepsis: Meets Sepsis-3 with compelling clinical/radiologic evidence of infection; cultures negative or non-sterile only.
- CRS + Sepsis (overlap): Meets ASTCT and Sepsis-3 criteria (definite or probable) with an independent source of infection.
- Indeterminate: Insufficient data to assign the above.

#### 3.6.2 Use of treatment response (supportive only)

Rapid improvement in fever/hemodynamics ≤12–24 h after tocilizumab/steroids will be considered supportive of CRS; improvement ≤48–72 h after targeted antibiotics/source control will be regarded as supportive of sepsis. These signals cannot override strong, conflicting microbiologic/radiologic evidence and will not be used as the sole criterion.

#### 3.6.3 For the primary analysis, we mapped adjudication to a binary reference

1. Sepsis-positive (=1): Definite/Probable Sepsis and CRS+Sepsis
2. CRS-positive (=0): Definite/Probable CRS
3. Excluded: Indeterminate
4. Predictor: logR = log[IL-6 (pg/mL)] − log[PCT (pg/mL)], using the index pre-treatment sample.
5. We will report the following:

- AUC (95% CI), sensitivity/specificity/PPV/NPV, LR+/LR−.
- Primary descriptive cut-off chosen by Youden’s J; dual thresholds define rule-out (target LR– ≤0.2) and rule-in (target LR+ ≥5) grey-zone in between.
- We will use two pre-set cut-offs for the IL-6/PCT ratio at each landmark (t_0_, +24 hours, +48 hours): rule-out targets sensitivity ≥0.90 and NPV ≥0.90; rule-in targets specificity ≥0.80 and PPV ≥0.80; values in between define the grey zone. All zones will be reassessed with the next daily IL-6/PCT (24–48 hours later). We will report how many grey-zone episodes move to rule-out or rule-in on repeat testing, how long that takes, and note any switches from rule-in or rule-out back to grey. For context, we will describe clinical features (day post–CAR-T, ANC group, SOFA, infections found, ICU use). The aim is to determine whether a simple repeat test can clarify unclear cases, without altering patient care in this study. We will present a small plot or table of daily zone transitions to D14.
- (Optional) PR curve and partial AUC at high sensitivity.

#### 3.6.4 Sensitivity analyses (robustness checks)

1. Re-map CRS+Sepsis to CRS: treat overlap cases as CRS (instead of sepsis) and re-run the ROC. If AUC stays similar, your result is robust to how overlaps are labelled.
2. Definite-only analysis: drop all “Probable” cases and keep only high-certainty “Definite” CRS/Sepsis. If performance holds, your findings aren’t driven by ambiguous cases.
3. Include Indeterminate as a third class (one-vs-rest ROC): instead of excluding them, test the biomarker’s ability to separate each class from the rest. This ensures that excluding uncertain cases does not bias the results.
4. Exclude samples taken after tocilizumab/steroids: these drugs can distort IL-6/PCT. Removing such samples checks that your accuracy doesn’t rely on post-treatment values.

### 3.7 Statistical considerations and data analysis

The statistical analysis plan will provide a comprehensive overview of the methodologies used to analyse the collected data and address the study objectives.

#### 3.7.1 Statistical methods

We will use blinded clinical adjudication (sepsis vs CRS) as the binary reference standard. The primary analysis evaluates the index pre-treatment (t_0_) IL-6/PCT ratio with ROC analysis to estimate AUC and 95% CIs and to derive rule-out and rule-in cut-offs. We will also compare the ratio against IL-6 and PCT alone, and perform a comparative time-series analysis (t_0_, 24 h, 48 h, and prespecified landmarks to day 14) to characterise temporal patterns and identify optimal diagnostic windows.

#### 3.7.2 Sample size and recruitment strategy

For the primary lymphoma cohort, we will target ~45–50 evaluable patients, over-recruiting by 10–15% to offset non-evaluable samples. We anticipate an AUC ≈of 0.80 vs 0.50 (null), yielding ~80% power (α=0.05) and AUC 95% CIs ≈ of ±0.10–0.15. We aim for ≥15–20 adjudicated sepsis episodes to ensure adequate precision; if accrual falls short, we will either extend recruitment within the predefined window or report wider exact 95% CIs and use bootstrap resampling (≥1,000 draws) for AUC and cut-off uncertainty.

Enrolment in the expansion indications (ALL, MM) will be opportunistic across sites with no per-site quotas. Recruitment will cease when (i) the lymphoma cohort reaches its target sample size or (ii) the recruitment window ends (8–12 months), whichever occurs first. Expansion enrolment is exploratory, with a pooled target of ~20–30 patients and a minimum acceptable pooled sample of ≥10. An optional ≤3-month grace period after lymphoma completion may be used solely to reach the expansion event threshold (e.g., ≥15 sepsis episodes; ideally ≥15 CRS). If this threshold is not met, recruitment will close, and expansion analyses will remain descriptive only.

#### 3.7.3 Secondary parsimonious model

We will pre-specify a limited multivariable logistic model (≤5 predictors in total) to estimate the independent contribution of log(IL-6/PCT) after routine covariates: day post-infusion (continuous, days since CAR-T at sampling), SOFA score at sampling (continuous, 0–24), ANC group (binary: <0.5 vs ≥0.5 ×10_9_/L, same-day complete blood count), and lactate (mmol/L, continuous). These capture timing, organ dysfunction, and neutropenia, which plausibly influence the probability of sepsis and PCT dynamics. To guard against overfitting in this small sample, we will use ridge penalisation as needed and bootstrap internal validation (≥1000 resamples) to report optimism-corrected AUC with 95% CIs. Firth’s correction will be applied if quasi-separation occurs. We will also report the incremental discrimination (ΔAUC) of the parsimonious model versus IL-6/PCT alone. If SOFA completeness is poor (e.g., >30% of episodes have partial SOFA), SOFA will be excluded from the model and retained descriptively only; the model will proceed with the remaining pre-specified covariates (day post-infusion, ANC group, lactate, and log[IL-6/PCT]). ANC (×10_9_/L) will be abstracted from the CBC closest to each IL-6/PCT draw (± 6–12 hours) and stored as a continuous value; for stratified analyses it will be categorised as <0.5 vs ≥0.5 ×10_9_/L.

#### 3.7.4 External validation and heterogeneity (secondary/exploratory)

Combined non-lymphoma expansion cohort (e.g., B-ALL and BCMA-myeloma pooled) will be analysed collectively under identical sampling/laboratory procedures. The lymphoma-derived IL-6/PCT cut-off will be applied unchanged. We will report ROC/AUC with 95% CIs (bootstrap, ≥1,000 resamples) and operating characteristics (sensitivity, specificity, PPV, NPV with exact CIs). If the combined cohort accrues <15 adjudicated sepsis episodes, results will be descriptive only (no regression/ROC inferences).

#### 3.7.5 Pooled mixed-effects analysis (conditional, secondary)

If the expansion cohort accrues ≥15–20 adjudicated sepsis episodes overall, we will fit an exploratory mixed-effects logistic model pooling the lymphoma and combined expansion cohorts (outcome: sepsis vs CRS), with random intercept for site and the following fixed effects: log(IL-6/PCT), group (lymphoma vs combined expansion), day post-infusion, SOFA at sampling, ANC <0.5×10_9_/L, and lactate. A group × log(IL-6/PCT) interaction will test for heterogeneity of biomarker effect. To mitigate overfitting, we will apply ridge penalisation if needed and perform bootstrap internal validation (≥1,000 resamples) to obtain optimism-corrected AUC (95% CIs). Sensitivity analyses will address class imbalance (e.g., inverse-probability weighting) and disease-stratified ROC. All pooled analyses are secondary/exploratory and do not alter the primary inference confined to the lymphoma cohort.

#### 3.7.6 IL-6/PCT ratio versus clinical outcomes

We will assess associations between the t_0_ IL-6/PCT ratio (index pre-immunomodulator sample) and the risks of ICU admission, vasopressor requirement, and 30-day all-cause mortality. The ratio will be log_2_-transformed (effect per doubling) and z-standardised. ICU/vasopressor outcomes will be analysed using mixed-effects logistic regression with a random intercept for site; mortality will be analysed using a Cox model with site-clustered robust standard errors. Models will adjust for prespecified covariates (age, sex, disease category, CAR-T product, lymphodepletion regimen). We will check non-linearity with restricted cubic splines and report adjusted odds ratios/hazard ratios with 95% CIs. Estimates are exploratory and intended to inform future multicentre power calculations.

#### 3.7.7 Descriptive statistics

Baseline demographics, microbiology, fever pattern, organ support, outcomes, and routine labs will be summarised as n (%) or median [IQR]. Between-group balance (CRS vs Sepsis) at baseline will be shown with standardised differences (no significance testing).

#### 3.7.8 Statistical software

All analyses will utilise Python (version 3.x) to ensure complete transparency, reproducibility, and cost efficiency. Using open-source tools allows for rigorous statistical modelling (e.g., logistic regression, ROC analysis) and code-based validation by independent reviewers. The significance level (p-value) will be set at <0.05 for all analyses. An independent statistician will thoroughly review the statistical procedures and studies to ensure accuracy and reliability.

#### 3.7.9 Missing data

We will report missingness by variable/timepoint. For primary analysis, missing IL-6 or PCT at t_0_ will be substituted with the following available sample (within 6 hours). For the secondary model, single imputation will use the cohort median for continuous variables (excluding indicator variables), with a complete-case sensitivity. Missing data for the IL-6/PCT vs. clinical outcomes analysis will be handled via multiple imputation (≈20 datasets), with bootstrap CIs (≥1,000 resamples) or Firth penalisation used if small-sample issues arise. When individual SOFA components are unavailable within the allowed window, they will be treated as missing (no imputation at the component level), and a partial SOFA will be reported with an incomplete indicator. Sensitivity analyses will: (i) assume missing components are normal (best-case), and (ii) exclude episodes with >2 missing components. Results will be compared to the primary analysis to assess robustness. For laboratory results reported as below the Limit of Detection/Quantification (LOD or LOQ), LOD/2 or LOQ/2 (primary analysis) will be substituted to permit log-transformations and ratio calculations. Sensitivity analyses will be repeated using LOD substitution. Values above the assay upper limit will be re-run with dilution per lab protocol. If a laboratory reports “<LOQ” but ≥LOD, we can treat it the same way (use LOQ/2) and show a sensitivity check.

### 3.8 Study outcomes

#### 3.8.1 Primary outcome

Diagnostic accuracy of IL-6/PCT for classifying adjudicated sepsis vs CRS in the R/R B-cell lymphoma cohort, summarised by ROC AUC with 95% CI (DeLong). We will also report sensitivity, specificity, and prespecified cut-offs (plus an *a priori* grey-zone if used). *Unit of analysis:* episode. *Time frame:* index episode within days 0–14 post-CAR-T infusion.

#### 3.8.2 Secondary outcomes (exploratory)

No multiplicity adjustment for secondary/exploratory outcomes; interpretations are hypothesis-generating.

##### 3.8.2.1 Parsimonious model accuracy

- Optimism-corrected AUC (95% CI) of the prespecified ≤5-predictor logistic model (predictors defined in Methods).
- Incremental discrimination ΔAUC (95% CI) vs IL-6/PCT alone.
- Prespecified variant excluding SOFA if >30% incomplete (report the same AUC and ΔAUC).

##### 3.8.2.2 External validation (non-lymphoma expansion cohort)

- AUC (95% CI; bootstrap) for IL-6/PCT using the unchanged lymphoma-derived cut-off.
- Operating characteristics (95% CIs): sensitivity, specificity, PPV, NPV at that fixed cut-off.
- If <15 adjudicated sepsis episodes accrue, results will be descriptive only (no inferential ROC/regression).

##### 3.8.2.3 Pooled exploratory accuracy and heterogeneity

- Optimism-corrected AUC (95% CI) from an exploratory mixed-effects logistic model pooling lymphoma and expansion cohorts.
- Heterogeneity of effect via group × log(IL-6/PCT) interaction (estimate and p-value).
- Disease-stratified ROC/AUC and class-imbalance sensitivity analyses (reported as exploratory).

##### 3.8.2.4 Temporal dynamics of biomarkers

- Longitudinal concentrations of IL-6, PCT, and the IL-6/PCT ratio during days 0–14 post-CAR-T, described and modelled (mixed-effects or equivalent).
- Trajectory summaries by CRS vs sepsis subgroup.
- Reclassification across landmarks (t_0_ → 24h → 48h): transitions from grey-zone → rule-out/rule-in and time to classification.
- Pre-register neutropenia stratified ROC

##### 3.8.2.5 Associations with short-term clinical outcomes (descriptive/exploratory; will not alter primary inference)

- Associations between biomarker profiles (absolute values and ratios) and: ICU admission, vasopressor requirement, 30-day mortality; report effect-size estimates (e.g., odds ratios or hazard ratios with 95% CIs) to inform future multicentre power calculations.

## Study limitations

We summarise key limitations of this exploratory study and the steps taken to mitigate their impact on interpretation.

1. Sample size and event imbalance: This exploratory study (≈45–50 evaluable patients) may yield wide CIs and limited power for subgroup effects, especially because sepsis is less frequent than CRS in the first 14 days. Mitigation: target ≥15–20 sepsis episodes, use bootstrap (≥1000) for optimism-corrected AUC/CIs, and include an independent validation/expansion cohort.
2. Cut-off optimism and external validity: Data-driven thresholds from the learning cohort may overestimate performance and may not generalise to other indications/sites. Mitigation: pre-specify rule-in/rule-out targets, fix the cut-off for external validation, and report validation metrics without recalibration.
3. Adjudication subjectivity: Differentiating CRS vs sepsis is imperfect and may introduce classification bias. Mitigation: two blinded adjudicators + third tie-breaker, calibration exercise, and Cohen’s κ with reasons for discordance; pre-specified sensitivity maps (overlap to sepsis/CRS; definite-only).
4. Treatment timing effects: Tocilizumab/steroids and antibiotics can shift IL-6/PCT dynamics even with pre-dose sampling. Mitigation: use pre-immunomodulator values for primary/landmark analyses, time-stamp antibiotics, and run timing sensitivity (t_0_ ≤6 h vs >6 h after antibiotics).
5. Missingness and SOFA completeness: Not all SOFA components are available at each draw; surrogates (SpO_2_/FiO_2_, AVPU mapping) may not perfectly replicate canonical measures. Mitigation: compute partial SOFA with an incomplete flag, pre-specified surrogates, and sensitivity (best-case; exclude >2 missing components); drop SOFA from the model if completeness is poor.
6. Assay/platform and site variability: Analytical differences and workflows across centres could affect absolute cytokine values and turnaround. Mitigation: ISO 15189 labs, harmonised pre-analytics, fixed platforms (Elecsys IL-6; BRAHMS PCT), Quality Control/Quality Assurance, and random intercept for site in pooled analyses.
7. Repeated measures and grey-zone dynamics: Daily sampling to Day 14 introduces correlated data and zone transitions that may confuse headline accuracy. Mitigation: restrict primary accuracy to t_0_ (with 24/48-h landmarks), treat later days as descriptive trajectories, and report reclassification rates transparently.
8. Observational design: Biomarker results do not guide care, so clinical impact is not tested here. Mitigation: this study is designed to estimate accuracy and inform a future prospective trial with predefined thresholds and decision pathways.

## Data Availability

The original contributions presented in the study are included in the article/supplementary material. Further inquiries can be directed to the corresponding author.
